# Cannabidiol-Loaded Mixed Polymeric Micelles of Chitosan/Poly(Vinyl Alcohol) and Poly(Methyl Methacrylate) for Trans-Corneal Delivery

**DOI:** 10.3390/pharmaceutics13122142

**Published:** 2021-12-13

**Authors:** Alejandro Sosnik, Ronya Ben Shabo, Hen Moshe Halamish

**Affiliations:** Laboratory of Pharmaceutical Nanomaterials Science, Department of Materials Science and Engineering, Technion-Israel Institute of Technology, Haifa 3200003, Israel; ronyabs22@gmail.com (R.B.S.); henmoshe@campus.technion.ac.il (H.M.H.)

**Keywords:** polymeric micelles, cannabidiol (CBD), spray-drying, ocular drug delivery, corneal epithelial cells

## Abstract

Ocular drug delivery is challenging due to the very short drug residence time and low permeability. In this work, we produce and characterize mucoadhesive mixed polymeric micelles (PMs) made of chitosan (CS) and poly(vinyl alcohol) backbones graft-hydrophobized with short poly(methyl methacrylate) blocks and use them to encapsulate cannabidiol (CBD), an anti-inflammatory cannabinoid. CBD-loaded mixed PMs are physically stabilized by ionotropic crosslinking of the CS domains with sodium tripolyphoshate and spray-drying. These mixed PMs display CBD loading capacity of 20% *w*/*w* and sizes of 100–200 nm, and spherical morphology (cryogenic-transmission electron microscopy). The good compatibility of the unloaded and CBD-loaded PMs is assessed in a human corneal epithelial cell line. Then, we confirm the permeability of CBD-free PMs and nanoencapsulated CBD in human corneal epithelial cell monolayers under liquid–liquid and air–liquid conditions. Overall, our results highlight the potential of these polymeric nanocarriers for ocular drug delivery.

## 1. Introduction

*Cannabis sativa* has emerged as a promising therapeutic agent to treat a broad spectrum of local and systemic diseases [1,2], and the global market is expected to grow at a compound annual growth rate of ~20% and reach USD 82.2 billion in 2027 [3]. Cannabidiol (CBD, Figure S1, see Supplementary Materials), one of the more than 120 phytocannabinoids produced by it [4], has neuroprotective, antiemetic, antioxidant, anti-cancer, and anti-inflammatory properties and it is not psychotropic [5]. In this context, it has been proposed in the therapy of cancer, epilepsy, pain, inflammation, and autism spectrum disorder with promising clinical results [1,2,6,7]. The development of CBD formulations is challenging because it is highly lipophilic (partition coefficient—log P—of 6.3) [8]; CBD is classified into Class II of the Biopharmaceutics Classification System (BCS) [9,10]. Oral CBD is often administered in oil or alcoholic formulations [11], and undergoes limited intestinal absorption and substantial hepatic first-pass, which results in a very low oral bioavailability (<20%) [12]. Low solubility also precludes CBD administration by other routes. 

Recently, CBD has been proposed in the treatment of different degenerative and inflammatory diseases of the eye [13,14,15,16,17] and the local administration of cannabinoids to the eye has been attempted with ophthalmic drops [18]. However, this delivery strategy is of limited efficacy for highly hydrophobic compounds such as CBD because solubility in the aqueous lachrymal medium is critical to ensure ocular absorption. The residence time of conventional liquid formulations on the eye surface is extremely short (15–30 s) owing to lacrimation [19]. In addition, the remaining drug has to cross very dense ocular tissues (e.g., cornea, conjunctiva) to reach the inner layers [20,21,22]. Thus, the bioavailability of drugs administered by using standard eye drops is ˂5% of the total administered dose. In this scenario, the use of advanced drug delivery systems for ocular delivery is called for.

The use of nanotechnology for the prophylaxis, diagnosis, and treatment of disease has been coined nanomedicine and it has provided valuable tools to improve the efficacy of a plethora of drugs [23,24] in adult disease [25,26], and to a more limited extent in pediatrics [27]. Following this trend, nanocarriers have become one of the most investigated strategies to improve diagnosis and treatment of ocular diseases because they can been engineered to mucoadhere to the eye surface and prolong the drug residence time, to transiently open epithelial tight junctions and cross the corneal epithelium by a paracellular pathway [28]. The diameter of the nanocarrier has to be in the 100–500 nm range to undergo transport across mucus, and its surface charge tuned to improve retention at the administration site [29]. The cornea displays negative surface charge owing to the nature of the mucus, making positively charged nanoparticles ideal for electrostatic interaction and prolonged residence time, which may also improve permeability into inner eye layers.

Polymeric micelles (PMs) are nanostructures formed by the aggregation of amphiphilic block or graft copolymers in aqueous medium above the critical micellar concentration (CMC) and display sizes between 10 and several hundreds of nanometers [30,31,32]. During self-assembly, the hydrophobic segments fold inwards and form the hydrophobic core that can be capitalized to encapsulate hydrophobic cargos and increase their apparent solubility in water [32]. Conversely, the hydrophilic segments fold outwards and generate the hydrophilic corona that physically stabilizes the aggregate in the dispersion medium and sometimes provides a barrier for controlled release of the cargo from the micellar core. The aggregation pattern, the shape, and the structure of the PM depend on the molecular weight (MW), the hydrophilic–hydrophobic balance (HLB), and the architecture of the copolymer molecule and changes in these molecular features may lead to different micellar structures, from the typical core-corona [32,33], to flower-like [34] and multimicellar ones [35]. PMs usually display good cell compatibility and biocompatibility and a highly functionalized surface area that allows an efficient interaction with surfaces and chemical modifications to actively target nanoencapsulated drugs to specific body sites [36]. PMs are capable of stabilizing poorly water-soluble drugs physicochemically, and they have been originally investigated to prolong the drug circulation time in the bloodstream after intravenous administration and target solid tumors by the enhanced permeation and retention effect [30]. We pioneered the application of PMs in mucosal drug delivery, including oral [37], ophthalmic [38], and intranasal administration routes [39], and stated some of the key features for the design of mucoadhesive counterparts [40]. In this framework, we produced mucoadhesive PMs by the hydrophobization of the side-chain of mucoadhesive hydrophilic polymeric backbones of chitosan (CS) and poly(vinyl alcohol) (PVA) with hydrophobic blocks such as poly(epsilon-caprolactone), poly(*N*-isopropyl acrylamide), and poly(methyl methacrylate) (PMMA) [41,42,43,44,45] and used them to produce mixed PMs with optimal cell compatibility and permeability across epithelium models in vitro [46]. We also introduced non-covalent crosslinking as a way to physically stabilize them and, at the same time, preserve the chemical structure of the cargo [42,43].

Aiming to improve the aqueous solubility of CBD and its transport across epithelial barriers, in this work, we initially produced and characterized mixed CS-*g*-PMMA/PVA-*g*-PMMA PM that load up to 20% *w*/*w* of CBD. Then, we assessed their cell compatibility and permeability across an in vitro model of human corneal epithelium.

## 2. Materials and Methods

### 2.1. Production of Mixed Chitosan-g-Poly(Methyl Methacrylate):Poly(Vinyl Alcohol)-g-Poly(Methyl Methacrylate) Polymeric Micelles

CS-*g*-PMMA was synthesized by the free radical polymerization of methyl methacrylate (MMA, Alfa Aesar, Heysham, UK) in water, as described elsewhere [43,44]. For this, low MW CS (0.4 g, degree of deacetylation of 94%; viscosity of ≤100 mPa.s, MW of ~50,000 g/mol, Glentham Life Sciences, Corsham, UK) was dissolved in nitric acid 70% (0.05 M in water, 100 mL, Bio-Lab Ltd., Jerusalem, Israel) that was degassed by sonication (30 min, Elmasonic S 30, Elma Schmidbauer GmbH, Singen, Germany). Then, a solution of tetramethylethylenediamine (0.18 mL, TEMED, Alfa Aesar, Sigma-Aldrich, St. Louis, MO, USA) in degassed water (50 mL) was poured into the CS solution and purged with nitrogen for 30 min, at RT. The purged CS solution was magnetically stirred, heated to 35 °C and 0.142 mL of purified MMA pre-treated with aluminum oxide (5 g, pore size 58 Å, ~150 mesh, Sigma-Aldrich, St. Louis, MO, USA) to remove the free radical inhibitor, was dispersed in degassed water (48 mL) and added to the reaction mixture. Finally, a solution of cerium (IV) ammonium nitrate (0.66 g, CAN, Strem Chemicals, Inc. Newburyport, MA, USA) in degassed water (2 mL) was added and the free radical polymerization reaction was allowed to proceed for 3 h at 35 °C under continuous N_2_ flow. After 3 h, the reaction was quenched by adding 0.13 g of hydroquinone (HQ, Merck GmbH, Hohenbrunn, Germany). The product was purified by dialysis against distilled water using a regenerated cellulose dialysis membrane with molecular weight cutoff (MWCO) of 12–14 kDa (Spectra/Por^®^ 4 nominal flat width of 75 mm, diameter of 48 mm and volume/length ratio of 18 mL/cm, Spectrum Laboratories, Inc., Rancho Dominguez, CA, USA) for 48–72 h and freeze-dried (Labconco Free Zone Plus 4.5 L Benchtop Freeze Dry System, Kansas City, MO, USA). The product was stored at 4 °C until use. This copolymer is named CS-PMMA30, where 30 represents the relative PMMA weight content in the copolymer (%PMMA), as determined by proton-nuclear magnetic resonance (^1^H-NMR, see below) [43,44]. The same chemical pathway was used for the synthesis of a PVA-*g*-PMMA copolymer with a %PMMA of 16%, namely PVA-PMMA16 [45]. For this, PVA (0.4 g, Mowiol^®^ 4–88, weight-average MW of 31,000 g/mol, 87–89% hydrolysis, Sigma-Aldrich, St. Louis, MO, USA) was dissolved in distilled water (100 mL) at RT, and TEMED (0.18 mL in 50 mL of degassed water) was dissolved in nitric acid 70% (0.45 mL). Then, TEMED and PVA solutions were degassed by sonication for 30 min, mixed and purged with nitrogen for 30 min, at RT. The solution was heated to 35 °C, and 0.142 mL of MMA pre-treated with aluminum oxide was dispersed in degassed water (48 mL) and added to the reaction mixture. Finally, a CAN solution (0.66 g in 2 mL of degassed water) was added, and the reaction allowed to proceed for 2 h at 35 °C. The reaction product was purified and dried as described above and stored at 4 °C.

Reaction yields were calculated according to Equation (1)

(1) $$\%\mathrm{Yield}\;=\frac{W_{\mathrm{dry}}}{W_{\mathrm{pol}}+{\;W}_{\mathrm{MMA}}\;}\times 100$$

where $W_{\mathrm{dry}}$ is the weight of dry copolymer (CS-PMMA30 or PVA-PMMA16) obtained after dialysis and freeze-drying, $W_{\mathrm{pol}}$ is the weight of the CS or PVA used in the reaction mixture, and $W_{\mathrm{MMA}}$ is the weight of MMA used in the reaction. All the weights are expressed in g.

For permeability studies in cell monolayers, PVA-PMMA16 was fluorescently labeled by the conjugation of fluorescein isothiocyanate (FITC, Sigma-Aldrich, St. Louis, MO, USA) and then, used to produce mixed PMs that contain a 1:1 weight ratio of CS-PMMA30 and PVA-PMMA16 (see below). Briefly, PVA-PMMA16 (100 mg) was dissolved in 2 mL of *N,N*-dimethylformamide (DMF, Bio-Lab Ltd., Jerusalem, Israel) under magnetic stirring. Then, FITC was dissolved in DMF (70 mg/mL, 0.2 mL), added to the copolymer solution and the mixture stirred for 16 h protected from light, at 32 °C. Finally, the product was dialyzed (48 h, regenerated cellulose dialysis membrane, MWCO of 3500 Da, Membrane Filtration Products, Inc., Seguin, TX, USA), freeze-dried (72–96 h), and stored protected from light at 4 °C until use.

^1^H-NMR spectra of CS-PMMA30 and PVA-PMMA16 were recorded in a 400 MHz Bruker^®^ Avance III High Resolution spectrometer (Bruker BioSpin GmbH, Rheinstetten, Germany) and analyzed with SpinWorks 4.0 software (University of Manitoba, Winnipeg, MB, Canada). For this, we used 5% *w*/*v* solutions in deuterated dimethyl sulfoxide (DMSO-*d6*, Cambridge Isotope Laboratories, Inc., Tewksbury, MA, USA). Chemical shifts are reported in ppm using the signal of DMSO (2.50 ppm) as internal standard. Pure CS was used as control and a solution prepared in 5% *w*/*v* deuterium oxide (D_2_O, Sigma-Aldrich, St. Louis, MO, USA) and trifluoroacetic acid (5% *v*/*v*, Sigma-Aldrich, St. Louis, MO, USA); CS is insoluble in DMSO. In this case, the signal of H_2_O (4.75 ppm) was used as internal standard.

To quantify the relative PMMA weight content in CS-PMMA30, a calibration curve of CS and MMA in D_2_O was built using physical mixtures with different MMA:CS weight ratio (0.5–10, R^2^ = 0.9502) that were dispersed in D_2_O with 5% *v*/*v* of trifluoroacetic acid, as reported elsewhere [43,44].

To quantify the %PMMA in PVA-*g*-PMMA16, a calibration curve of MMA:PVA (weight ratio of 0.5–6.25, R^2^ = 0.9978) was built in DMSO-*d6* and the ratio between the integration of the characteristic signals of PVA and the methyl group of MMA calculated [45].

The thermal behavior of the copolymers, pure CBD, and the different CBD-free and CBD-loaded PMs was analyzed by differential scanning calorimetry (DSC) in a 2 STAR^e^ system simultaneous thermal analyzer with STAR^e^ Software V13 (Metter-Toledo, Schwerzenbach, Switzerland) with intra-cooler (Huber TC100) under dry N_2_ flow (20 mL/min) and In as standard. Samples (5.0–15.0 mg) were sealed in 40 µL Al-crucibles pans (Metter-Toledo, Schwerzenbach, Switzerland) and subjected to different heating–cooling cycles (10 °C/min). For CS-PMMA30, PVA-PMMA16, and the CBD-free and CBD-loaded mixed PMs, the protocol was the following: (i) cooling from 35 to −70 °C; (ii) heating from −70 to 100 °C to erase the thermal history; (iii) isothermal heating at 100 °C (30 min) to eliminate water traces; (iv) heating from 100 to 300 °C, and (v) cooling from 300 to 35 °C. For pure CBD, the protocol was: (i) cooling from 35 to −70 °C; (ii) heating from −70 to 100 °C; (iii) isothermal heating at 100 °C (30 min), and (iv) cooling from 100 to 35 °C. The glass transition temperature (T_g_) and the melting temperature (T_m_) of copolymers and CBD-free and CBD-loaded PMs were determined in the first heating ramp. Melting enthalpy (ΔH_m_) values were normalized to the polymer weight content in the copolymer sample.

CBD-free and CBD-loaded mixed CS-*g*-PMMA30:PVA-*g*-PMMA16 (1:1 weight ratio) PMs were prepared by using a microfluidics system designed and fabricated in our laboratory [47]. The microfluidics chip is composed of three layers: the upper and lower layers are glass-made, and the middle layer is made of a 700 μm width p-type silicon wafer (100) upon which the microfluidic channels were embedded. The width and depth of the channels are 500 μm. The lengths of the inlet and outlet channels in the Y-shape are 10 and 8 mm, respectively. To clean the microfluidic system before use, two continuous infusion pumps (Laboratory Syringe Pump, SYP-01, MRC, Kfar Saba, Israel) were filled with 10 mL of ethanol (Gadot, Netanya, Israel), pumped, and refilled again with 10 mL of distilled water that was also pumped. For the preparation of the mixed PMs (1:1 weight ratio between the graft copolymers), 16 mg CS-PMMA30 was dissolved in water (1 mL) supplemented with 10 µL of glacial acetic acid (pH of 5.5, Gadot, Netanya, Israel). In parallel, 8 mg of PVA-PMMA16 was dissolved in absolute methanol (1.5 mL, Gadot, Netanya, Israel). Then, CBD (4 mg, THC Pharm GmbH, Offenbache, Germany) was dissolved in the PVA-PMMA16 methanol solution. Both solutions (CS-PMMA30 in water and CBD/PVA-PMMA16 in methanol) were magnetically stirred at RT for at least 1 h, and then mixed in 1.5:0.5 methanol:water volume ratio. After 10–15 min of additional magnetic stirring at RT, this solution (1 mL) and distilled water (9 mL) were loaded into two separate syringes and pumped at flow rates of 0.1 and 0.9 mL/min, respectively. The final concentration of each copolymer in the final volume was 0.04% *w*/*v* and the final CBD concentration was 0.02% *w*/*v*, rendering mixed PMs containing 20% *w*/*w* of CBD. For crosslinking of the mixed PMs, sodium tripolyphosphate (TPP, Sigma-Aldrich, St. Louis, MO, USA) was added to the water phase (2–3 µL of 1% *w*/*v* TPP solution per 1 mL of mixed PM suspension). CBD-free PMs were prepared by using the same method, though without the addition of CBD, and used as blank.

The different mixed PMs were collected in glass vials and immediately characterized by dynamic light scattering (DLS, see below). Then, they were frozen at −80 °C and freeze-dried (Labconco Free Zone Plus 4.5 L Benchtop Freeze Dry System, Kansas City, MO, USA) or, conversely, spray-dried (Nano Spray Dryer Büchi B-90 HP, Büchi Labortechnik AG, Flawil, Switzerland) using an open loop configuration that is feasible for aqueous systems, inlet temperature 110 °C, 80% spraying, 30 mbar pressure, 112 kHz frequency, 90% feeding rate, and 125 L/min airflow rate, yield of 45%. Dry products were stored at 4 °C until use.

### 2.2. Characterization of Mixed Chitosan-g-Poly(Methyl Methacrylate):Poly(Vinyl Alcohol)-g-Poly(Methyl Methacrylate) Polymeric Micelles

The hydrodynamic diameter (D_h_, expressed by intensity), the polydispersity index (PDI, a measure of the size distribution), and the zeta-potential (Z-potential, an estimation of the surface charge density) of 0.1% *w*/*v* PMs (fresh, and dried and redispersed) were characterized by DLS (Zetasizer Nano-ZS, Malvern Instruments, Malvern, UK) operating at a scattering angle of 173°. Each value is expressed as mean ± standard deviation (S.D.) of at least three independent samples, while each DLS or Z-potential measurement is an average of at least seven runs.

The quantification of the number of PMs per mL of suspension and the visualization of their Brownian motion was conducted by nanoparticle tracking analysis (NTA, NanoSight^®^ NS500-Zeta HSB system with high sensitivity camera, Malvern Instruments, Malvern, UK) under scattering mode. PMs were diluted 2–100 times in the same medium used to prepare them to fit the measurement range of the instrument (10^7^–10^9^ particles per mL) and immediately measured. Experimental concentrations (particle per mL) were corrected by the dilution factor.

The morphology of the fresh CBD-free (blank) and CBD-loaded mixed PMs (0.1% *w*/*v*) before and after crosslinking was visualized by cryogenic-transmission electron microscopy (cryo-TEM). Mixed PMs were vitrified in a controlled environment vitrification system (CEVS). For this, ~3 µL of the sample was placed on a carbon-coated perforated polymeric film placed on a TEM grid (200 mesh), mounted on tweezers, and the sample turned into a thin film (thickness <300 nm) by blotting the excess of the solution with a filter paper-covered metal strip. The grid was plunged into liquid ethane and transferred and kept in liquid N_2_. Samples were visualized in a FEI Talos 200C High Resolution TEM (Thermo Fisher Scientific, Waltham, MA, USA) equipped with a cryo-holder (cryo-specimen holder, Gatan, Inc., Pleasanton, CA, USA) at −181 °C and an accelerating voltage of 120 kV.

The morphology of freeze-dried and spray-dried mixed PMs after redispersion was visualized by high resolution-scanning electron microscopy (HR-SEM, acceleration voltage of 2–4 kV, Ultraplus, Zeiss, Oberkochen, Germany). Samples were placed on top of a silicon wafer (cz polished silicon wafers <100> oriented, highly doped N/Arsenic, SHE Europe Ltd., Livingston, UK). Next, the wafer was attached to the grid using carbon-tape and additional tape was placed on its frame. At the corners of the frame silver paint (SPI# 05002-AB-Silver, SPI supplies, West Chester, PA, USA) was applied. Images were obtained without carbon coating by using In-lens detector at 3–4 mm working distance. Samples of freeze- and spray-dried mixed PMs were also analyzed by HR-SEM.

CBD after dissolution and spray-drying was quantified by the Beam test [48,49]. Briefly, a solution of 5% *w*/*v* KOH (Bio-Lab Ltd., Jerusalem, Israel) in absolute ethanol (Gadot, Netanya, Israel) was prepared and kept at RT until use. Then, the corresponding sample for analysis was dissolved in absolute ethanol, 5% *w*/*v* KOH solution (~250 μL) was added, and the sample stirred for 5 min to allow the oxidation of the extracted CBD to HU-331 and bis-HU-331. Then, the absorbance was measured at 274 nm in a Multiskan GO Microplate Spectrophotometer (Thermo Fisher Scientific Oy, Vantaa, Finland), and interpolated in a calibration curve of CBD in absolute ethanol with concentrations between 0.0001% and 0.1% *w*/*v* (R^2^ = 0.9992), treated with 5% *w*/*v* KOH and the CBD concentration calculated. Results are expressed as mean ± S.D. of at least three independent measurements.

### 2.3. Compatibility of Mixed Polymeric Micelles in Human Corneal Epithelial Cells

The cell compatibility of the different mixed PMs was evaluated in the human corneal epithelial (hCEc) cell line HCE-2 [50.B1] (ATCC^®^ CRL-11135™, ATCC^®^, Manassas, VA, USA). Cells were seeded on 75 cm^2^ flasks pre-coated with a mixture of fibronectin (0.01 mg/mL, Biological Industries, Migdal HaEmek, Israel), bovine collagen type I (0.03 mg/mL, Thermo Fisher Scientific, Waltham, MA, USA), and bovine serum albumin (0.01 mg/mL, Sigma-Aldrich, St. Louis, MO, USA) in RPMI-1640 medium (Sigma-Aldrich, St. Louis, MO, USA). Cells were cultured in keratinocyte-serum free medium (Gibco Laboratories, Grand Island, NY, USA), supplemented with bovine pituitary extract (0.05 mg/mL, Gibco Laboratories, Grand Island, NY, USA), epidermal growth factor (5 ng/mL, Gibco Laboratories, Grand Island, NY, USA), hydrocortisone (500 ng/mL, Merck Millipore, Burlington, MA, USA), human recombinant insulin (0.005 mg/mL, Gibco Laboratories, Grand Island, NY, USA), and penicillin/streptomycin (5 mL of a commercial mixture of 100 U/mL de penicillin and 100 μg/mL streptomycin per 500 mL medium, Sigma-Aldrich, St. Louis, MO, USA), maintained at 37 °C in a humidified 5% CO_2_ atmosphere and split every 6–7 days. Cells were harvested by trypsinization (trypsin-EDTA 0.25%, Sigma-Aldrich, St. Louis, MO, USA) and the number of live cells quantified by the trypan blue (0.4%, Sigma-Aldrich, St. Louis, MO, USA) exclusion assay. To determine the cell compatibility of the mixed PMs, cells were seeded and cultured in pre-coated 96-well plates (1.0 × 10^4^ cells/well) and allowed to attach (96 h). The sample preparation method was as follows: 1% *w*/*v* PMs freshly prepared and freeze- or spray-dried and redispersed in phosphate buffered saline (PBS, Sigma-Aldrich, St. Louis, MO, USA), were diluted in culture medium to a final concentration of 0.07%, 0.1%, and 0.15% *w*/*v* and incubated overnight at 37 °C. Then, the culture medium was replaced by 200 µL of micellar suspension. After 4 and 24 h, the medium was removed and fresh medium (100 μL) and sterile 3-(4,5-dimethylthiazol-2-yl)-2,5-diphenyltetrazolium bromide solution (25 μL, MTT, 5 mg/mL, Sigma-Aldrich, St. Louis, MO, USA) was added. Samples were incubated for 4 h (37 °C, 5% CO_2_), the supernatant was removed, the formazan crystals dissolved with DMSO (100 μL, Bio-Lab Ltd., Jerusalem, Israel), and the absorbance measured at 530 nm (with reference wavelength of 670 nm) in a Multiskan GO Microplate Spectrophotometer. The percentage of live cells was estimated with respect to a control treated only with culture medium (and considered 100% viable). Since the dilutions can affect the amount of nutrients available for the cells, the culture medium of the control cells was also diluted 6.67 times in PBS to mimic the most diluted PM suspension (0.15% *w*/*v*).

### 2.4. Permeability of Mixed Polymeric Micelles across a Human Corneal Epithelium Model In Vitro

A permeability assay was conducted with mixed crosslinked PMs in hCEc monolayers, an in vitro model of the human cornea [50]. For this, FITC-labeled mixed CS-PMMA30:PVA-PMMA16 PMs (a stock 0.1% *w*/*v* dispersion prepared in water supplemented with acetic acid of pH 5.5) were diluted in transport medium (Hank’s Balanced Salt Solution, HBSS, Sigma-Aldrich, St. Louis, MO, USA) buffered to pH 7.4 with 4-(2-hydroxyethyl)-1-piperazineethanesulfonic acid (25 mM, HEPES, Sigma-Aldrich, St. Louis, MO, USA) and NaHCO_3_ (0.35 g/L, Sigma-Aldrich, St. Louis, MO, USA) to the final concentration (0.01% and 0.03% *w*/*v*). For this, mixed PMs were prepared with a CS-PMMA30:FITC-labeled PVA-PMMA16:PVA-PMMA16 weight ratio of 1:0.4:0.6. For non-covalent crosslinking, a 1% *w*/*v* TPP solution in HBSS was prepared and 3 µL of TPP solution per 1 mL of PM dispersion was added at least 6 h before the permeability experiment. Fluorescently labeled mixed PMs that were spray-dried and redispersed in HBSS were also used.

Experiments were performed 14–21 days post-seeding of hCEc (4.55 × 10^5^ cells/well) on cell culture inserts (ThinCert™, culture surface of 113.1 mm^2^, 3.0 µm pore size, Greiner Bio-One GmbH, Frickenhausen, Germany) maintained in 12-well plates (15.85 mm diameter, 16.25 mm height, Greiner CELLSTAR, Monroe, NC, USA) with 0.5 and 1.5 mL of keratinocytes serum-free medium (see above for culture of hCEc), to the apical and basolateral, respectively. The culture medium was replaced every 2–3 days and the integrity of the hCEc monolayer was characterized by transepithelial electrical resistance (TEER) measurements performed with an epithelial volt-ohm-meter EVOM2 (WPI, Sarasota, FL, USA).

The permeability assay was conducted in two corneal epithelium models with air–liquid (AL) and liquid–liquid (LL) interface. Eight days after culture, inserts were lifted to produce an AL culture or left in culture medium for a LL one and cultured for 1–2 more weeks. Only inserts where the TEER was >140 Ω·cm^2^ were used. At the beginning of the experiment, the medium in the apical (0.5 mL) and basolateral (1.5 mL) compartments was replaced with HBSS without PMs and the cells incubated for 20 min at 37 °C. Then, the transport medium in the donor (apical) compartment was replaced by the sample (0.4 mL) containing the PMs and in the acceptor (basolateral) compartment by fresh transport medium (1.2 mL). Plates were incubated on an orbital shaker (MRC Ltd., Holon, Israel) at 37 °C and gently shaken to minimize the impact of the unstirred water layer. After 5, 10, 15, 30, 45, 60, 90, 120, 180, and 240 min, medium (600 μL) was extracted from the acceptor compartment and replaced by the same volume of fresh transport medium to maintain the total volume constant. The extracted medium was used for quantification of the transported PMs by fluorescence spectrophotometry in a Fluoroskan Ascent Plate Reader (Thermo Fisher Scientific Oy, Vantaa, Finland) using black 96-well flat bottom plates (Greiner Bio-One, Kremsmunster, Austria) at wavelengths of 485 nm for excitation and 538 nm for emission according to a calibration curve of non-crosslinked and crosslinked PMs in HBSS in a concentration range between 0.0001% and 0.1% *w*/*v* (R^2^ = 0.9916 and 0.9913, respectively). The apparent permeability (P_app_) was calculated according to Equation (2)

(2) $$P_{\mathrm{app}}=\frac{\mathrm{dc}}{\mathrm{dt}}\cdot \frac{1}{A\cdot C_{0}}\;\left[\mathrm{cm}\cdot s^{-1}\right]$$

where dc/dt is the permeability rate of the PMs (expressed in μg/s) across the monolayer, $C_{0}$ is the initial concentration of the PMs in the donor compartment (expressed in $\mu g/{\mathrm{cm}}^{3}$), and A is the surface area of the membrane (1.131 cm^2^). Results are expressed as mean ± S.D. of at least three independent experiments.

At the end of each experiment (240 min), 50 μL was also removed from the donor compartment to calculate the mass balance by fluorescence spectrophotometry (see above). The percentage of PMs retained by the cell monolayer (%PMs inside cells) was estimated according to Equation (3)

(3) %PMs inside cells=100%−%Final donor−%Final acceptor

where 100% is the total percentage of PMs at the beginning of the experiment, %Final donor is the percentage of PMs that remained in the donor compartment at the end of the experiment (4 h), and %Acceptor is the percentage of PMs that crossed to the acceptor compartment during the experiment until the end time point of 4 h. Results are expressed as mean ± S.D. of at least three independent experiments.

We also measured the permeability of CBD-loaded PMs by tracking the gradual increase of the CBD concentration in the acceptor compartment over time by the Beam test (see above). For this, fresh CBD-loaded mixed PMs were prepared as described above in HBSS instead of water without any fluorescence labeling of the PMs. Briefly, 0.1% *w*/*v* CBD-loaded PMs (20% *w*/*w* loading) were diluted to a final total copolymer concentration of 0.03% *w*/*v* (CBD final concentration of 20% *w*/*w* in the PMs), and the permeability assay was conducted under AL and LL setups. After 5, 10, 30, 60, 90, 120, 180, and 240 min, 600 μL of medium was extracted from the acceptor compartment and replaced by the same volume of fresh transport medium to maintain the total volume in the chamber constant. The extracted medium was used for quantification of the transported CBD-loaded PMs by diluting them with ethanol (2 mL) to extract CBD and conducting the Beam test for which a CBD calibration curve in ethanol in a concentration range between 0.0001% and 0.1% *w*/*v* (R^2^ = 0.9992) was built. At the end of the experiment (240 min), 50 μL was also removed from the donor side of each sample to calculate the mass balance, as described above. Results are expressed as mean ± S.D. of at least three independent experiments.

### 2.5. Statistical Analysis

Statistical analysis of the different experiments was performed by *t*-test on raw data (Microsoft Excel, Microsoft Office 2019, Microsoft Corp., Seattle, WA, USA). *p*-values of less than 0.05 were considered as statistically significant.

## 3. Results and Discussion

### 3.1. Rationale

Mucoadhesive polymers have been investigated in the development of advanced local and trans-mucosal drug delivery systems [29]. CS is a polycationic natural polysaccharide of glucosamine repeating units with pendant primary amine groups that are protonated at pH < 5.8 [51]. CS promotes mucoadhesion in different mucosal tissues including the corneal epithelium [52,53]. In addition, CS favors the absorption of nanoparticles by the paracellular route by transiently opening tight junctions in different epithelia [54], including the corneal epithelium [55]. CS-*N*-acetylcysteine (Lacrimera^®^, CROMA, Leobendorf, Austria) eye drops have been developed as a medical device and clinically trialed for the treatment of dry eye with promising results [56]. PMs can also cross epithelia by transcellular mechanisms [57]. CS emerged as a promising component to develop amphiphilic nanoparticles (e.g., PMs) that exploit this pathway [43]. CS slowly degrades in the biological tissues, mainly by the activity of lysozyme and human chitinases [58].

PVA is a synthetic water-soluble polymer broadly used in a wide range of food, biomedical, and pharmaceutical applications with very good biocompatibility [59]. PVA contains hydroxyl groups that enable the interactions with the mucin by H bonding [29]. The mucoadhesive strength of PVA is 5.11 N/cm^2^ [60], and greater than that of CS (0.58 N/cm^2^) [54].

CS is a polycation under physiological conditions, and cell toxicity has been associated with its electrostatic interaction with the cell membrane. However, its cell compatibility can be improved upon partial neutralization of the positive charges by ionotropic crosslinking with polyanionic molecules (e.g., TPP), by decreasing the degree of deacetylation or by chemically modifying or mixing it with other polymers that reduce the availability of cytotoxic free primary amine groups on the nanoparticle surface [61,62]. We improved the compatibility of amphiphilic CS-*g*-oligo(*N*-isopropylacrylamide) and CS-*g*-PMMA PMs in different cell types by crosslinking them ionotropically [35,43], and by developing mixed amphiphilic counterparts with PVA-*g*-PMMA [46]. These mixed nanoparticles capitalize on advantageous properties of both hydrophilic components such as mucoadhesion, opening of epithelial tight junctions, good encapsulation capacity of hydrophobic PMMA blocks for different hydrophobic cargos and cross epithelial models in vitro [46,63].

In this conceptual framework, we anticipated the potential of mixed CS-*g*-PMMA and PVA-*g*-PMMA PMs produced by the co-micellization to encapsulate CBD for trans-corneal delivery. The use of PMMA was supported by its broad use as pharmaceutical excipient in oral drug delivery systems [64,65] and in permanent biomedical implants such as intraocular lenses, bone cements, and fixation and other orthopedic devices [66,67,68]. Short PMMA blocks bound to hydrophilic backbones (e.g., CS, PVA) are expected to undergo excretion by renal filtration.

### 3.2. Production and Characterization of CBD-Loaded Mixed Polymeric Micelles

We synthesized CS-*g*-PMMA and PVA-*g*-PMMA copolymers by the free radical graft polymerization of MMA onto the backbone of both CS and PVA, respectively (Figure S2, see Supplementary Materials). Copolymers were analyzed by ^1^H-NMR (Figure S3, see Supplementary Materials) and the %PMMA calculated using calibration curves [43,44,45] was 30% and 16%, respectively, and copolymers named CS-PMMA30 and a PVA-PMMA16. Calculated yields were ~70% and ~75% for CS-PMMA30 and PVA-PMMA16, respectively.

Thermal analysis of the two graft copolymers used to produce the mixed PMs was conducted to compare the properties of CBD before and after nanoencapsulation. Pure CS and PVA were analyzed for comparison. Pure CS exhibited an exothermic peak starting at 230 °C due to thermal decomposition (Figure S4, see Supplementary Materials), in good agreement with the literature [69,70]. The T_g_ and T_m_ of PVA was detected at 78 and 196 °C (ΔH_m_ = 28.8 J/g), respectively [71]. When the graft copolymers were analyzed, CS-PMMA30 exhibited only one exothermic peak of CS decomposition at ~240 °C and PVA-PMMA16 displayed T_g_ at 87 °C and T_m_ at 179 °C (ΔH_m_ = 24.2 J/g). In both copolymers, the thermal transition of PMMA blocks could not be detected (Figure S4, see Supplementary Materials).

PMs can be produced by different methods such as simple dissolution and organic solvent diffusion and evaporation [72]. In this work, mixed CS-PMMA30:PVA-PMMA16 PMs with a 1:1 weight ratio were prepared by a solvent casting method in a microfluidics device [47] and their D_h_, PDI, Z-potential, and concentration in suspension were measured by DLS and NTA, at 25 and 37 °C. In addition, NTA was used to visualize their Brownian motion in suspension. All the mixed PMs, regardless of crosslinking and CBD loading or not, showed a monomodal size distribution (only one size population could be observed), as exemplified in Figure S5 (see Supplementary Materials) for 0.1% *w*/*v* CBD-free crosslinked mixed PMs. The D_h_ of the PMs ranged between 96 ± 6 nm for CBD-free non-crosslinked and 151 ± 8 nm for CBD-loaded crosslinked ones (Table 1). Temperature changes had a negligible effect on the micellar size. Generally, non-crosslinked PMs were smaller than the crosslinked counterparts, suggesting that the ionotropic crosslinking results in an enlargement of the PMs by bridging them. Crosslinking also resulted in the reduction of the Z-potential because the charge of the amine groups is partially neutralized by TPP. When comparing non-crosslinked with crosslinked PMs, crosslinking only very slightly reduces the Z-potential from +38 to +33–35 mV, without dramatic changes in the PDI value (Table 1). These findings indicated that the aggregation pattern was not dramatically affected by the crosslinking. Moreover, PMs showed a slight size growth upon CBD nanoencapsulation though without affecting the PDI or the surface charge. CBD-free mixed TPP-crosslinked PMs with CS-PMMA30:PVA-PMMA16 PMs weight ratios of 0.9:1.1 and 0.8:1.2 were also prepared and their size measured by DLS. No size or PDI changes could be observed. Since the goal of the graft copolymers combination was to improve the cell compatibility of the PMs by reducing the CS concentration on the surface, while preserving their ability to undergo ionotropic crosslinking with TPP and to transiently open tight junctions in epithelia that require CS, we continued the work only with PMs produced with a 1:1 weight ratio.

NTA was used to measure the D_h_ and concentration of the PMs and visualize them in suspension. According to NTA, the D_h_ of the PMs ranges between 100 and 180 nm. The same changes in the size of the PMs upon crosslinking or CBD encapsulation were observed by this technique. At 37 °C, the size of the PMs measured by NTA increased, along with a slight decrease in the PM concentration. This behavior might stem from a slightly different co-micellization pattern of the copolymers at this T, and especially in the presence of CBD. This size growth upon heating could not be detected by DLS, which exhibits lower size resolution than NTA [73]. Representative snapshots of the visualization of different PMs in suspension by NTA are presented in Figure S6 (see Supplementary Materials).

CBD can degrade upon exposure to temperature >37 °C and especially light [74]. CBD could also undergo auto-oxidation [75]. For example, Δ^9^- and Δ^8^-tetrahydrocannabinol impurities were detected in pure CBD samples stored in darkness at RT for three months [76]. Since CBD is highly lipophilic, it is usually dissolved in oily solvents that might undergo oxidation and trigger its oxidation.

**Figure 1:**
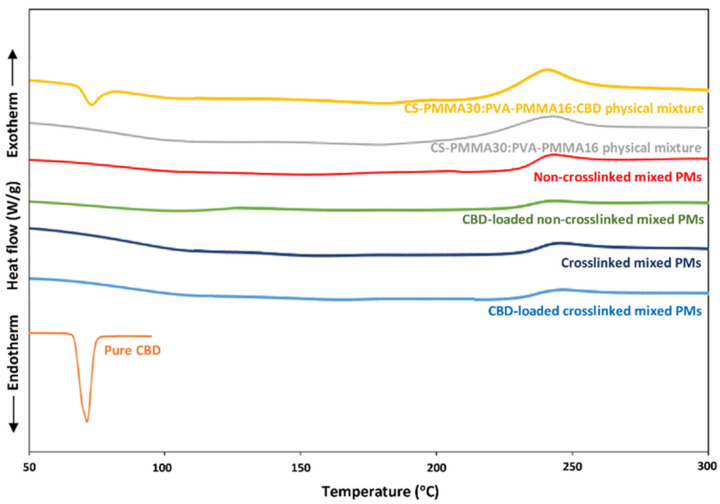
DSC thermograms of pure CBD, physical mixtures of copolymers without and with CBD and CBD-free and CBD-loaded spray-dried mixed PMs, as measured by DSC. The weight ratio in the CS-PMMA30:PVA-PMMA16 and CS-PMMA30:PVA-PMMA16:CBD physical mixtures was 1:1 and 1:1:0.5, respectively.

DSC is useful to characterize the crystallinity or amorphousness of pure drugs and polymers, and their combinations. We comparatively assessed the thermal properties of pure CBD, and CBD-free and CBD-loaded mixed PMs. Pure CBD showed T_m_ at ~70 °C (ΔH_m_ = 68.3 J/g) [77], while CS-PMMA30:PVA-PMMA16 physical mixtures (1:1 weight ratio) without and with 20% *w*/*w* CBD showed a broad exothermal peak in the 240–270 °C range associated with CS decomposition (Figure 1). In addition, the T_m_ of crystalline CBD in the physical mixture could be detected.

We also analyzed the thermal behavior of mixed PMs (non-crosslinked and crosslinked and CBD-free and CBD-loaded) after spray-drying. Since the crosslinking with TPP is not covalent, we did not expect to observe major differences between non-crosslinked and crosslinked PMs by DSC. The DSC thermogram of mixed PMs was a combination of those of the individual components, though the T_m_ of PVA was not detected in the mixed PMs, indicating that it is amorphous (Figure 1).

Drugs nanoencapsulated within hydrophobic polymeric nanoparticles are usually amorphous owing to the formation of solid solutions. The absence of a CBD melting endotherm in CBD-loaded PMs confirmed that this compound is amorphous (Figure 1). As mentioned above, CBD could undergo thermal degradation. Since some mixed PMs were spray-dried at relatively high temperature (105 °C), we wanted to ensure that the encapsulated CBD does not undergo thermal decomposition. The Nano Spray Dryer B-90 HP is expected to minimize the exposure of active molecules to the heat to a fraction of a second and thus, prevent their thermal decomposition [78]. However, the stability of each compound might change. Therefore, we dissolved pure CBD in absolute ethanol, at the final concentration obtained after the encapsulation process (0.02% *w*/*v*) and spray-dried it under the same conditions used for the mixed PMs. Then, spray-dried pure CBD was collected, and Beam test was conducted on three samples. The CBD content in the spray-dried samples was 98.5%, indicating that CBD withstands the spray-drying conditions.

**Figure 2:**
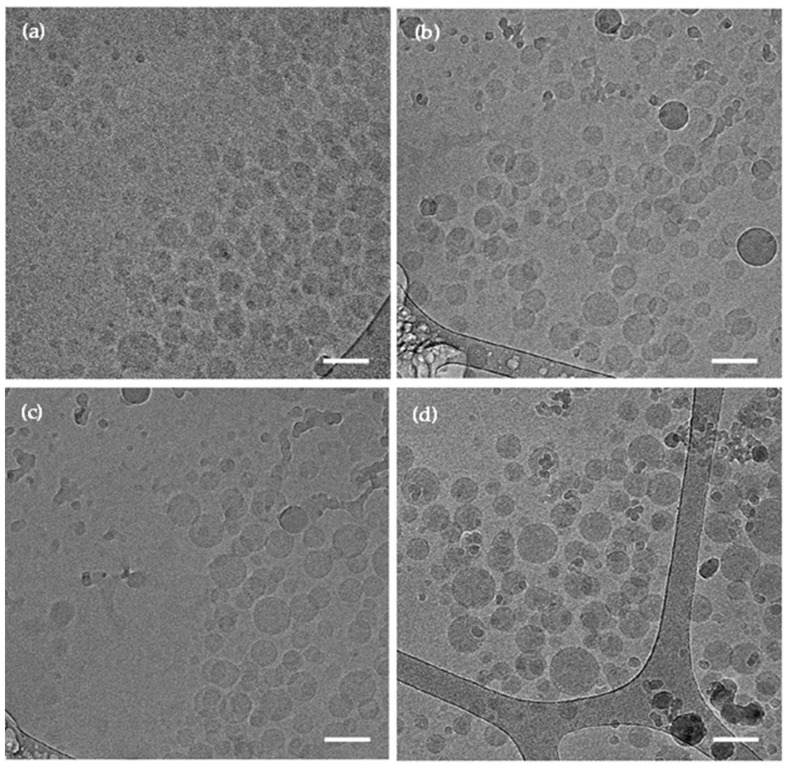
Representative cryo-TEM micrographs of fresh (**a**) CBD-free non-crosslinked mixed PMs, (**b**) CBD-free crosslinked mixed PMs, (**c**) CBD-loaded non-crosslinked mixed PMs, and (**d**) CBD-loaded crosslinked mixed PMs. Scale bar: 100 nm.

The spherical morphology of the CBD-free and CBD-loaded mixed PMs was visualized by cryo-TEM (Figure 2). Non-crosslinked mixed PMs showed a smaller diameter (70–90 nm) than crosslinked ones (100–120 nm for CBD-free PMs and 120–170 nm for CBD-loaded PMs). As expected, the diameter was smaller than the D_h_ measured by DLS and NTA because the latter methods measure the hydrodynamic size that comprises also hydration water.

### 3.3. Drying and Redispersion of CBD-Loaded Mixed Polymeric Micelles

Drying methods such as freeze-drying have been investigated to improve the long-term physicochemical stability of nanoformulations. In the case of the freeze-drying of nanoparticles (the most popular in the pharmaceutical industry), the addition of cryo/lyoprotectants in relatively large relative amounts is often required to preserve their original size upon redispersion [79]. Otherwise, nanoparticles might undergo irreversible agglomeration that prevents redispersion. In recent years, spray-drying has gained interest because additives are not needed [78]. In this context, CBD-free and CBD-loaded mixed PMs before and after crosslinking were freeze-dried and spray-dried and the morphology of the dry powders analyzed by HR-SEM. In addition, dry powders were redispersed in the original volume in water and characterized again by DLS.

**Figure 3:**
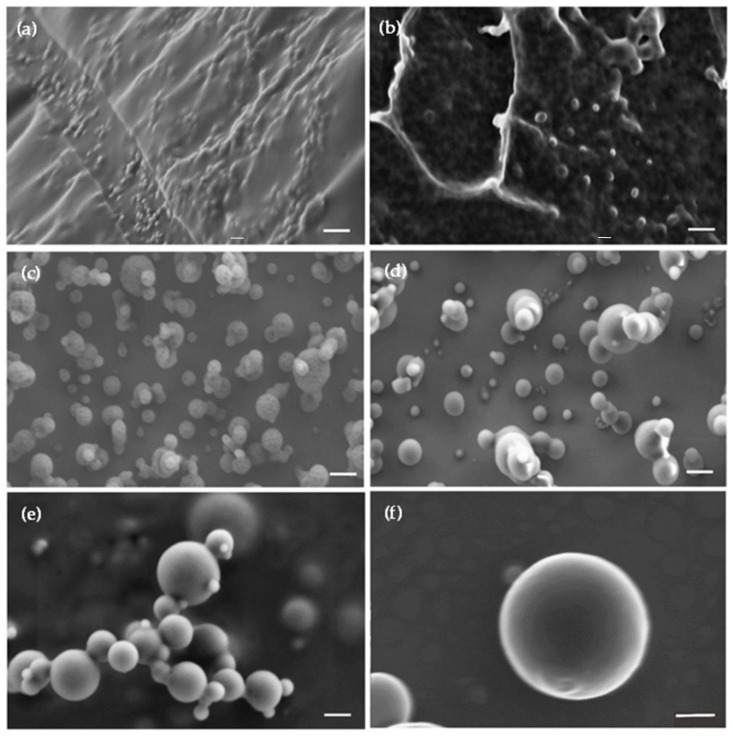
Representative HR-SEM micrographs of (**a**,**b**) freeze- and (**c**–**f**) spray-dried mixed polymeric micelles. (**a**) CBD-free non-crosslinked mixed PMs, (**b**) CBD-loaded non-crosslinked mixed PMs, (**c**) CBD-free non-crosslinked mixed PMs, (**d**) CBD-free crosslinked mixed PMs, (**e**) CBD-loaded non-crosslinked mixed PMs, and (**f**) CBD-loaded crosslinked mixed PMs. Scale bar: 200 nm.

Freeze-dried non-crosslinked mixed PMs were rounded and in the nanometer scale range (Figure 3).

However, as usual after freeze-drying of nanoparticles made of polysaccharides such as CS, they formed a polymeric network in which PMs are strongly bound to each other and they could not be easily visualized (Figure 3a). No major differences were observed upon CBD encapsulation (Figure 3b). When the PMs were spray-dried, powders showed spherical individual particles with smooth surface (Figure 3c–f). Some of the particles were larger than the size measured for the PMs by DLS, NTA, and cryo-TEM (up to 200 nm), and some of them were even in the micrometer scale range. These large particles are not the mixed PMs. Conversely, they are particles generated by the fast drying of the suspension droplets generated by the spraying head of the spray-dryer [78]. After freeze- and spray-drying, powders were resuspended in the original volume of water to render a final copolymer concentration of 0.1% *w*/*v*, and the D_h_ and PDI of the PMs was measured by DLS. Results are summarized in Table 2.

The redispersion of the freeze-dried mixed PMs was inefficient, the suspension was not completely translucent, and relatively large aggregates were observed by the naked eye. Only after at least 16 h of intensive magnetic stirring, the suspension became more translucent. DLS results indicated that although the D_h_ did not dramatically change (generally, a growth of 10–20 nm of the size was observed), the PDI values increased sharply, this phenomenon being more noticeable for CBD-loaded mixed PMs (from approximately 0.20 up to 0.73), meaning that these PMs form stable aggregates upon freeze-drying (Table 2). Conversely, resuspensions of spray-dried PMs were translucent even after only 2–3 h of magnetic stirring and no large aggregates could be visualized with the naked eye. The D_h_ and PDI of these PMs remained almost unchanged and PDI values were smaller than those of freeze-dried counterparts. Based on these results, cell studies in vitro were conducted with fresh and spray-dried redispersed CBD-free and CBD-loaded mixed PMs.

### 3.4. Compatibility of Mixed Polymeric Micelles with Human Cornea Epithelial Cells

**Figure 4:**
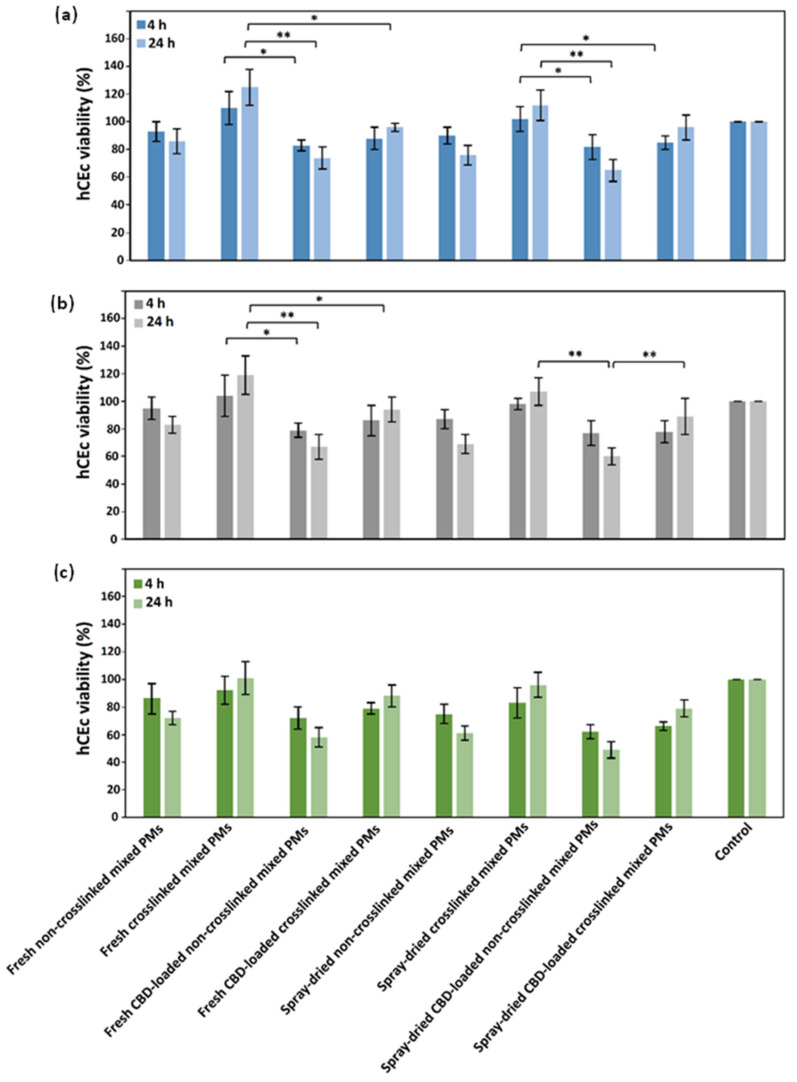
hCEc viability upon exposure to (**a**) 0.05%, (**b**) 0.10%, and (**c**) 0.15% *w*/*v* mixed PMs after 4 and 24 h, as estimated by the MTT assay (*n* = 5). * Statistically significant difference (*p* < 0.05); ** statistically significant difference (*p* < 0.01).

We evaluated the compatibility of CBD-free and CBD-loaded mixed PMs in a human cornea epithelial cell line. Cells were exposed to fresh and spray-dried PMs (0.05%, 0.10%, and 0.15% *w*/*v*) for 4 and 24 h and the viability estimated by the MTT assay. The lowest PM concentration (0.05% *w*/*v*) showed viability values ˃70% except for spray-dried and redispersed CBD-loaded non-crosslinked PMs that exhibited 65 ± 8% after 24 h (Figure 4a).

Cell viability values ˃70% comply with the guidelines of ISO 10993-5 for the evaluation of the cytotoxicity of medical devices in vitro. The cell viability loss increased for 0.10% *w*/*v* PMs, especially for the non-crosslinked ones due to the more positively charged surface that contributes to cell toxicity. In contrast, crosslinked PMs showed viability values ˃70%, regardless of the CBD loading and the spray-drying processing (Figure 4b). When the PM concentration increased to 0.15% *w*/*v*, the cell viability dropped more, especially for non-crosslinked samples that showed 62–72% viability, with fresh mixed PMs displaying higher viability values than the spray-dried ones (Figure 4c). Viability values ˃100% could be explained by cell stress upon exposure to the PMs, which results in a transient increase of the metabolic activity after 4 h with a decrease below 100% later (24 h). The viability decrease between fresh and spray-dried and redispersed PMs was not statistically significant. Thus, this processing method is a promising strategy to improve the physicochemical stability of the cargo and the PMs in the long-term [78]. Additionally, as discussed above, ionotropic crosslinking of CS domains in the mixed PMs not only was meant to physically stabilize them under extreme dilution but it also improved cell viability by 10–20% when compared to non-crosslinked counterparts, this phenomenon being independent of the CBD loading and the processing by spray-drying (or not). This trend was observed at all the PM concentrations, and particularly after 24 h. In addition, CBD consistently results in slightly lower viability values (viability decrease of ~10% after 4 h and ~20–30% after 24 h) than cells treated with unloaded PMs. The cytotoxicity CBD on human cells has been reported in the scientific literature though in most cases CBD was dissolved in a cosolvent such as ethanol or DMSO [80]. Interestingly, all 0.05% *w*/*v* and most 0.10% *w*/*v* PMs showed viability values that comply with the ISO 10993-5 guidelines (Figure 4a,b). Based on these findings, permeability assays with hCEc were conducted with 0.01% and 0.03% *w*/*v* crosslinked PMs to ensure optimal cell viability and the monolayer integrity for at least 4 h (see below). Non-crosslinked Pms were not sued because these concentrations are below the CMC of both copolymers [43,44,45].

### 3.5. Permeability of Mixed Polymeric Micelles across a Model of Corneal Epithelium In Vitro

Since the self-assembly of CS-PMMA30 and PVA-PMMA16 to form mixed PMs is random, the use of FITC-labeled PVA-PMMA16 in their preparation ensured the fluorescent labeling of all the PMs and preserved free amine groups in the sidechain of CS for ionotropic crosslinking with TPP and interaction and transient opening of tight junctions in epithelial monolayers.

**Figure 5:**
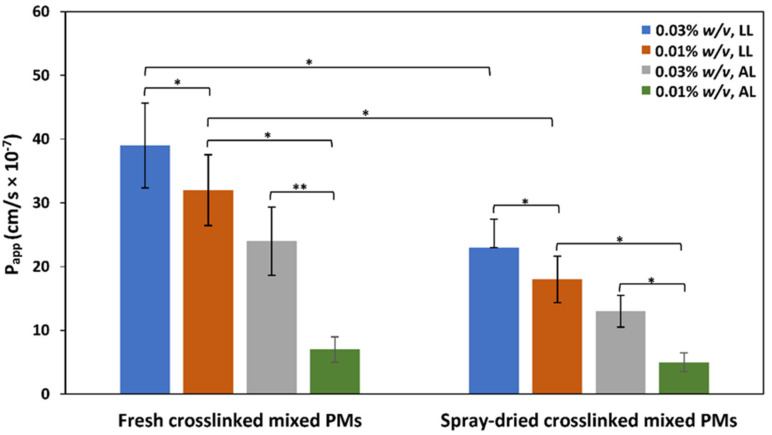
Apparent permeability coefficient (P_app_) of 0.01% and 0.03% *w*/*v* CBD-free crosslinked mixed PMs in hCEc monolayers under LL and AL conditions (*n* = 5). * Statistically significant difference (*p* < 0.05); ** statistically significant difference (*p* < 0.01).

First, we characterized the permeability of CBD-free mixed PMs in a corneal epithelium model by measuring the P_app_ under LL and AL conditions [81]. For these experiments, we utilized only crosslinked PMs because they are prepared at a concentration (0.1% *w*/*v*) above the CMC, crosslinked and then, diluted to final concentrations of 0.01% and 0.03% *w*/*v*, which are below the CMC of these copolymers. Non-crosslinked PMs were not used because they could disassemble upon dilution. To estimate the integrity of hCEc monolayers, TEER values were measured over 14–21 days until a constant value between 140 and 170 Ω·cm^2^ was measured for LL and AL conditions, respectively. An increase in the TEER value of 25–30 Ω·cm^2^ under AL incubation conditions on the same experiment day was consistent with the formation of stronger tight junctions by these epithelial cell lines, which might reduce the nanoparticle permeability across it. This comparison is physiologically relevant because the outer cells of the human corneal epithelium are exposed to air and covered by a thin layer of mucus that preserves their hydration state, what usually results in the formation of more and stronger tight junctions [81]. This behavior is like other epithelia exposed to air such as the nasal epithelium [46]. As expected, PMs systematically crossed the cell monolayer faster and the calculated P_app_ was greater for LL than for AL interface owing to the formation of stronger tight junctions by the latter model, as supported by TEER measurements (Figure 5). This performance was independent of the PMs (fresh or spray-dried and redispersed). For example, under LL conditions, fresh 0.03% crosslinked mixed PMs displayed P_app_ of 39 ± 7 × 10^−7^ cm/s (Figure 5). The same PMs under AL setup showed a P_app_ value of 24 ± 5 cm/s. Interestingly, spray-drying and redispersion led to a significant decrease of the P_app_ across hCEc monolayers when compared to fresh PMs, regardless the monolayer properties and the micellar concentration. For example, fresh 0.01% *w*/*v* crosslinked PMs under LL conditions displayed P_app_ of 24 ± 4 × 10^−7^ cm/s, while the spray-dried and redispersed counterparts of 18 ± 4 × 10^−7^ cm/s, values decreasing for AL conditions. In addition, the P_app_ was affected by the PM concentration and, in general, P_app_ values in hCEc monolayers under LL and AL conditions increased with the PM concentration, suggesting the key contribution of the paracellular pathway (Figure 5). Moreover, P_app_ values measured in hCEc were substantially smaller than measured for the same mixed PMs under the same conditions in an in vitro model that mimics the intestinal epithelium (Caco-2 cell line, P_app_ between 40 and 110 × 10^−7^ cm/s) [82] and the nasal epithelium (RPMI 2650, P_app_ between 34 and 60 × 10^−7^ cm/s) [46]. These differences in permeability in vitro are most probably associated with the differential ability of epithelial cells to form tight junctions [83].

At the end of each permeability study, we measured the fluorescence in both the donor and the acceptor chamber to calculate the mass balance and estimate the percentage of CBD-free mixed PMs retained by the cell monolayer due to unspecific adsorption or cell internalization, this percentage being always <12%, in good agreement with results reported elsewhere [84]. Based on this, intracellular trafficking is expected to have minimum effect on the permeability results.

After assessing the permeability of CBD-free PMs, we characterized the permeability of 0.03% *w*/*v* CBD-loaded fresh crosslinked mixed PMs containing 20% *w*/*v* CBD loading under LL and AL conditions. To evaluate the permeability of CBD encapsulated within mixed PMs, we did not label the PMs with FITC because CBD was extracted from the samples and the concentration measured by the Beam test (see above).

**Figure 6:**
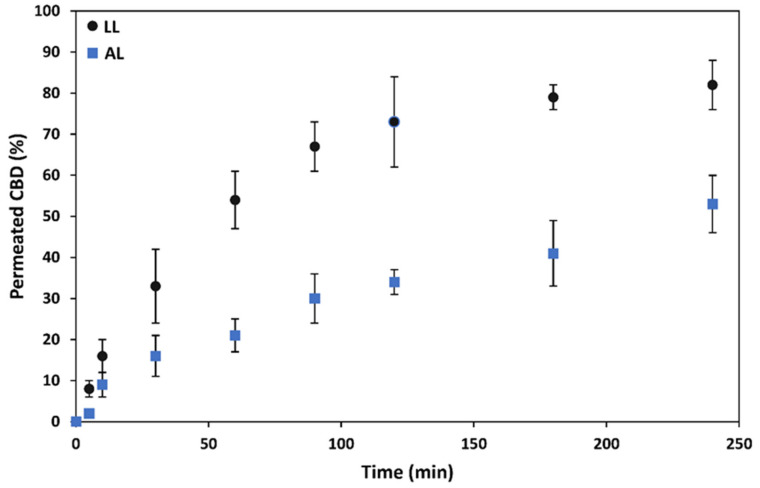
Permeability of 0.03% *w*/*v* CBD-loaded crosslinked mixed PMs across hCEc monolayer under LL and AL conditions (*n* = 3). The CBD loading in the PMs was 20% *w*/*w* and the final concentration in the donor compartment 0.006% *w*/*v*.

Results of these experiments indicated that fresh CBD-loaded crosslinked mixed PMs cross this model of corneal epithelium in vitro. As expected, the permeability rate under LL conditions was faster than under AL ones due to the formation of stronger tight junctions in the latter. For example, at the end of the permeability assay (4 h), 82% and 53% of the encapsulated CBD was measured in the acceptor compartment for LL and AL setups, respectively (Figure 6). These results of permeability rate were like those shown by unloaded PMs (data not shown) and indicated the key role played by the nanocarrier in the transport of these hydrophobic cargos across this biological barrier. The conduction of a similar permeability study with free CBD was not possible owing to its extremely low aqueous solubility (~10 μg/mL); the initial CBD concentration in the donor compartment by using the mixed PMs was 0.006% *w*/*v* (equivalent to 60 μg/mL).

It is also important to stress that our model of corneal epithelium is deprived of the outer mucus layer that covers the cornea. Our PMs are mucoadhesive and this feature is expected to prolong their residence time with respect to non-mucoadhesive ones, which in turn, could increase the amount of drug delivered across the cornea over time. As mentioned above, CBD-loaded crosslinked mixed PMs are larger than the unloaded ones though still in the size range that fits trans-mucosal paracellular transport. Overall, our results support the promise of these CBD-loaded nanocarriers as a drug delivery platform to the cornea and the inner eye.

## 4. Conclusions

This work reports on the synthesis and characterization of mucoadhesive mixed CS-PMMA30:PVA-PMMA16 PMs (1:1 weight ratio) for the loading of CBD and trans-corneal delivery of CBD for potential application in inflammatory eye conditions. A high CBD loading of up to 20% *w*/*w* was achieved by using a simple and reproducible microfluidic system. Mixed PMs showed monomodal size and narrow size distributions with sizes in the 100–170 nm range and spherical morphology, as visualized by cryo-TEM. In addition, mixed PMs were successfully stabilized physically by non-covalent crosslinking and the size of crosslinked counterparts increase slightly though it remained <200 nm, fitting mucosal drug delivery. Then, the cell compatibility of the different PMs was evaluated by using a human corneal epithelial cell line that serves as a model of outer surface of the eye. CBD-free and CBD-loaded mixed PMs showed good cell compatibility before and after the crosslinking. Additionally, we investigated the ability of the mixed PMs to cross models of epithelium in vitro and measured the P_app_ of the mixed PMs in confluent hCEc monolayers under LL and AL conditions. Permeability across corneal epithelium is a crucial step to ensure efficient ocular drug delivery. Findings showed that these mixed PMs cross this corneal epithelium model even under AL conditions, at which cells form stronger tight junctions most probably by transiently opening them (paracellular mechanism). Finally, these results were supported by showing the efficient permeability of CBD across both LL and AL models. Overall, our results support the promise of these PMs as a drug delivery platform for the treatment of eye diseases.

## Figures and Tables

**Table 1:** Hydrodynamic diameter (D_h_), polydispersity index (PDI), Z-potential and concentration of CBD-free and CBD-loaded mixed PMs (0.1% *w*/*v*) before and after TPP crosslinking, as measured by DLS and NTA.

Sample	Temperature (°C)	DLS			NTA	
		D_h_–Intensity (nm) ± S.D.	PDI ± S.D.	Z-Potential (+mV) ± S.D.	D_h_ (nm) ± S.D.	Concentration (×10^9^ Particles/mL)± S.D.
Non-crosslinked mixed PMs	25	100 ± 10	0.30 ± 0.07	+38 ± 2	114 ± 3	9.5 ± 0.3
	37	96 ± 6	0.32 ± 0.07	+33 ± 3	133 ± 1	3.9 ± 0.3
Crosslinked mixed PMs	25	140 ± 20	0.38 ± 0.05	+34 ± 4	129 ± 2	9.7 ± 0.4
	37	140 ± 10	0.31 ± 0.07	+32 ± 4	144 ± 4	3.6 ± 0.2
CBD-loaded non-crosslinked mixed PMs	25	144 ± 6	0.21 ± 0.02	+35 ± 2	151 ± 1	9.6 ± 0.1
	37	142 ± 9	0.19 ± 0.02	+33 ± 1	196 ± 2	3.4 ± 0.2
CBD-loaded crosslinked mixed PMs	25	147 ± 9	0.19 ± 0.03	+34 ± 3	120 ± 4	9.0 ± 0.7
	37	151 ± 8	0.17 ± 0.02	+33 ± 3	177 ± 4	3.9 ± 0.1

**Table 2:** Hydrodynamic diameter (D_h_) and polydispersity index (PDI) of CBD-free and CBD-loaded mixed PMs (0.1% *w*/*v*) before and after crosslinking that were freeze- or spray-dried and redispersed in the original volume of water, as determined by DLS at 25 °C.

Sample	Drying Method	D_h_–Intensity (nm) ± S.D.	PDI ± S.D.
Non-crosslinked mixed PMs	Freeze-drying	113 ± 2	0.33 ± 0.02
	Spray-drying	140 ± 10	0.41 ± 0.04
Crosslinked mixed PMs	Freeze-drying	139 ± 4	0.36 ± 0.01
	Spray-drying	135 ± 4	0.38 ± 0.03
CBD-loaded non-crosslinked mixed PMs	Freeze-drying	130 ± 10	0.73 ± 0.03
	Spray-drying	129 ± 6	0.30 ± 0.03
CBD-loaded crosslinked mixed PMs	Freeze-drying	155 ± 8	0.62 ± 0.03
	Spray-drying	142 ± 1	0.41 ± 0.06

## Data Availability

The data presented in this study are available upon request from the corresponding author.

## Supplementary Materials

The following are available online at https://www.mdpi.com/article/10.3390/pharmaceutics13122142/s1, Figure S1: Chemical structure of cannabidiol (CBD); Figure S2. Synthetic pathway of CS-PMMA30 and PVA-PMMA16 copolymers by the free radical graft polymerization of MMA. The reaction time was 3 and 2 h for the synthesis of CS- and PVA-based copolymers, respectively; Figure S3. ^1^H-NMR spectra of pure CS (in D_2_O), pure PMMA, pure PVA, CS-PMMA30 and PVA-PMMA16 (in DMSO-*d6*); Figure S4. Thermal analysis of pristine polymers and graft copolymers, as measured by DSC; Figure S5. Size distribution plot of 0.1% *w*/*v* CBD-free crosslinked mixed PMs, as measured by DLS; Figure S6. Representative NTA snapshots of (a) non-crosslinked mixed PMs, (b) crosslinked mixed PMs, (c) CBD-loaded non-crosslinked mixed PMs, and (d) CBD-loaded crosslinked mixed PMs, at 37 °C under scattering mode. The CBD loading is 20% *w*/*w*.
