# Supplementary material for: Cannabidiol-Loaded Mixed Polymeric Micelles of Chitosan/Poly(Vinyl Alcohol) and Poly(Methyl Methacrylate) for Trans-Corneal Delivery
Source: Pharmaceutics. 2021 Dec 13;13(12):2142. doi: 10.3390/pharmaceutics13122142 (PMC8703866; doi:10.3390/pharmaceutics13122142)
Supplement: Supplementary file 1 [file pharmaceutics-13-02142-s001.zip › pharmaceutics-1492957-supplementary.pdf]

pharmaceutics

Supplementary Materials: Cannabidiol-Loaded Mixed Polymeric Micelles of Chitosan/Poly(Vinyl Alcohol) and Poly(Methyl Methacrylate) for Trans-Corneal Delivery

Alejandro Sosnik, Ronya Ben Shabo and Hen Moshe Halamish

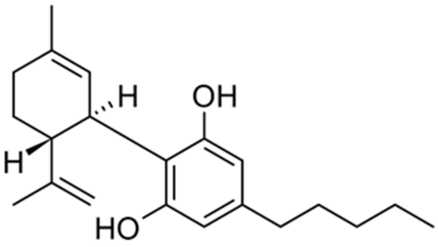

Figure S1. Chemical structure of cannabidiol (CBD).

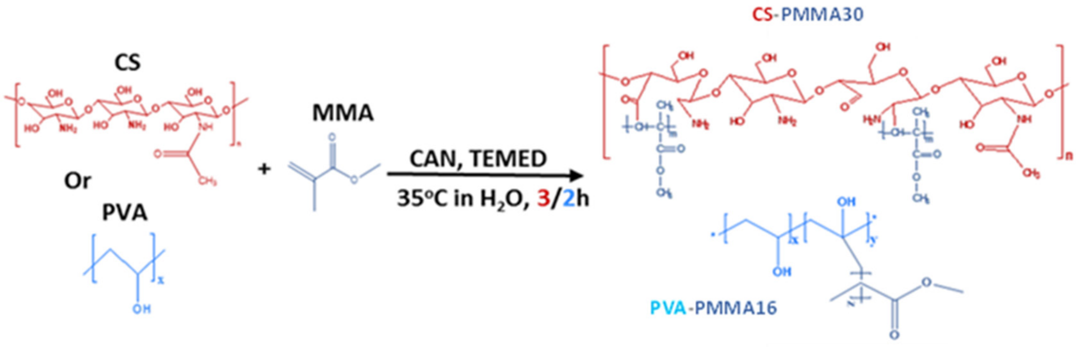

Figure S2. Synthetic pathway of CS-PMMA30 and PVA-PMMA16 copolymers by the free radical graft polymerization of MMA. The reaction time was 3 and 2 h for the synthesis of CS- and PVA-based copolymers, respectively.

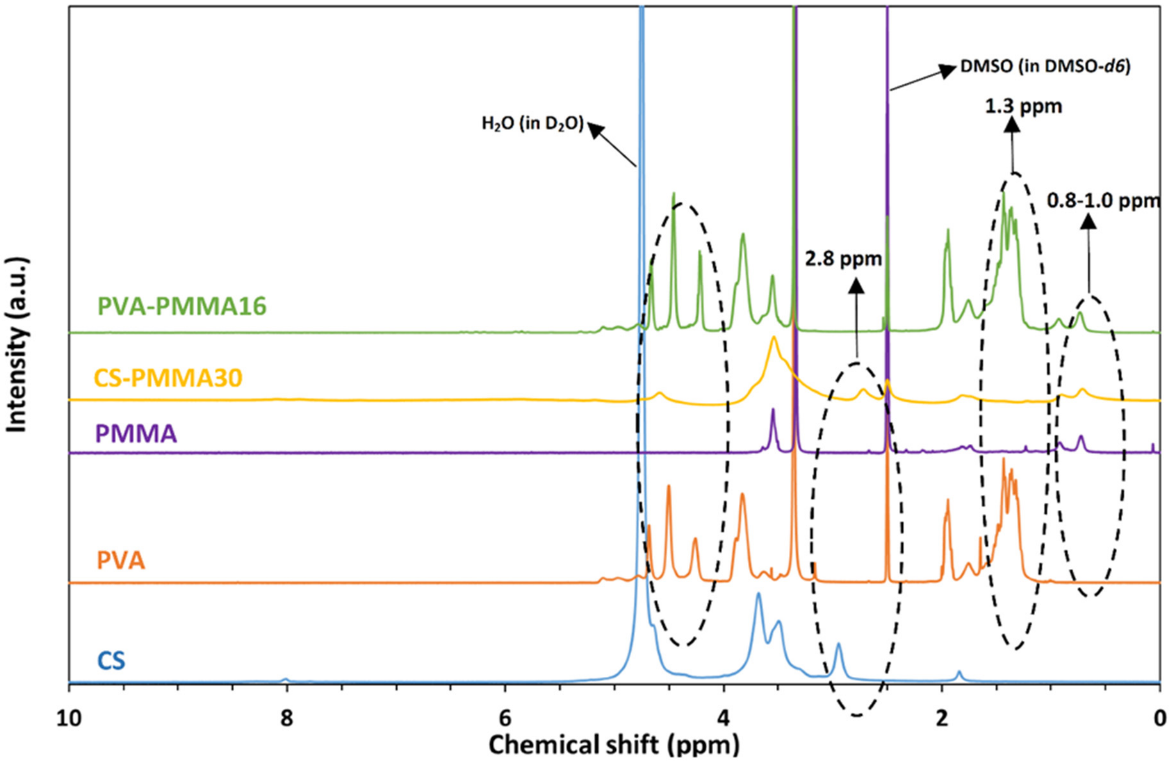

Figure S3. ^{1}H-NMR spectra of pure CS (in D_2O), pure PMMA, pure PVA, CS-PMMA30 and PVA-PMMA16 (in DMSO-*d6*).

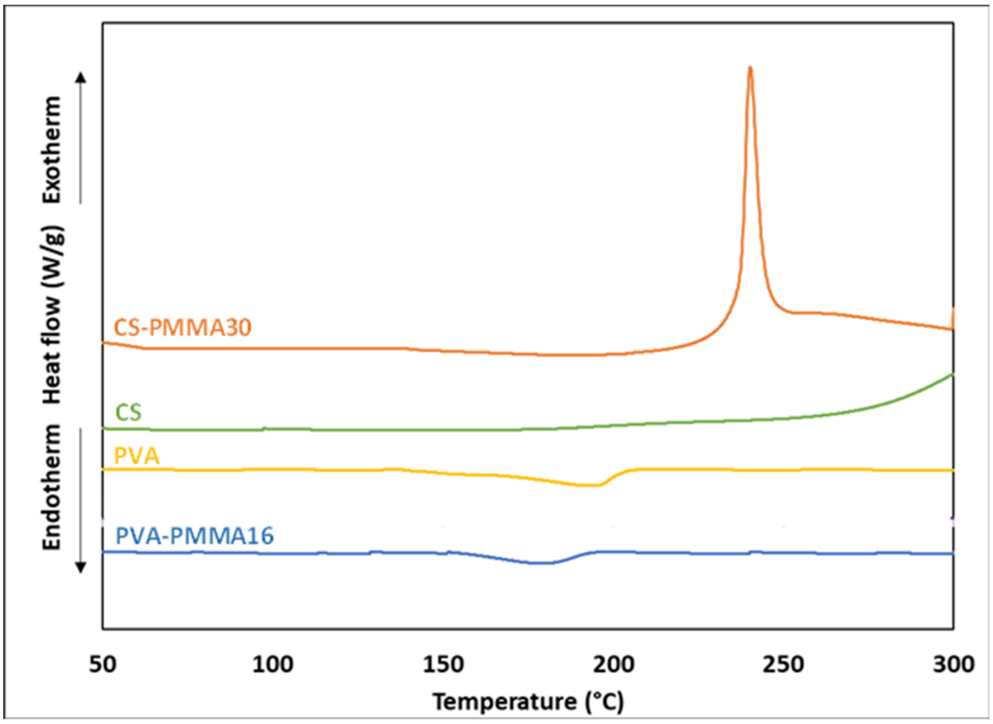

Figure S4. Thermal analysis of pristine polymers and graft copolymers, as measured by DSC.

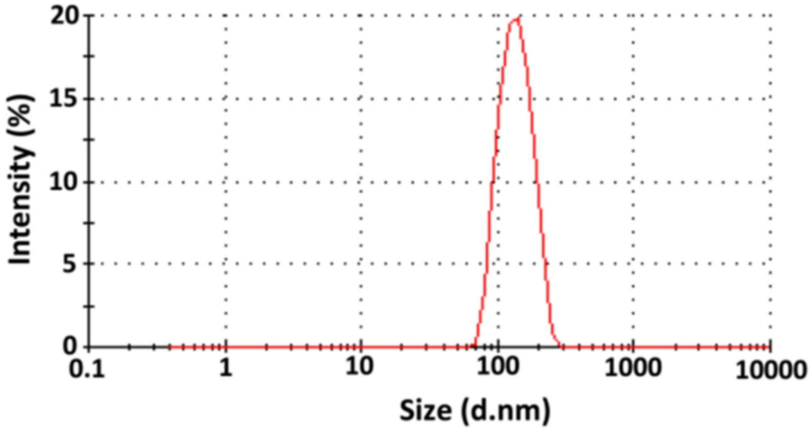

Figure S5. Size distribution plot of 0.1% *w*/*v* CBD-free crosslinked mixed PMs, as measured by DLS.

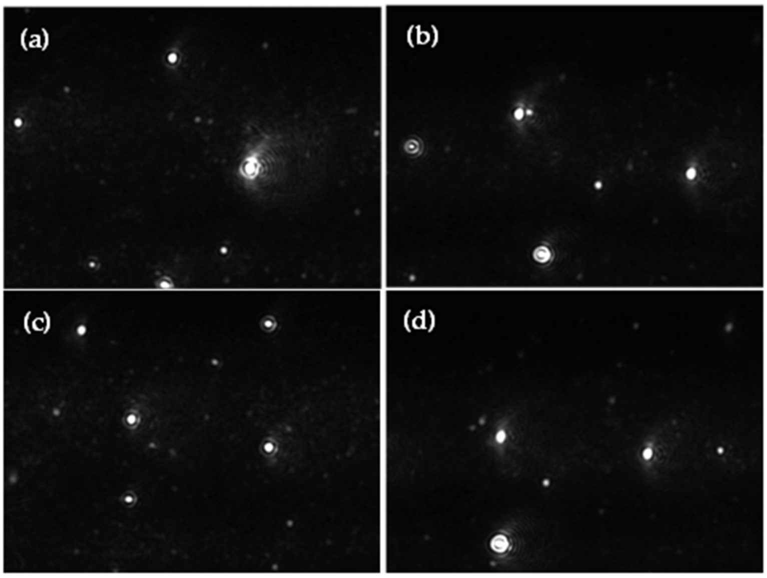

Figure S6. Representative NTA snapshots of (**a**) non-crosslinked mixed PMs, (**b**) crosslinked mixed PMs, (**c**) CBD-loaded non-crosslinked mixed PMs, and (**d**) CBD-loaded crosslinked mixed PMs, at 37 °C under scattering mode. The CBD loading is 20% *w*/*w*.
